# Mutation of cysteine 46 in IKK-beta increases inflammatory responses

**DOI:** 10.18632/oncotarget.5567

**Published:** 2015-09-10

**Authors:** Ting Li, Vincent Kam Wai Wong, Zhi Hong Jiang, Shui Ping Jiang, Yan Liu, Ting Yu Wang, Xiao Jun Yao, Xiao Hui Su, Feng Gen Yan, Juan Liu, Elaine Lai-Han Leung, Xiao Qin Yi, Yuen Fan Wong, Hua Zhou, Liang Liu

**Affiliations:** ^1^ State Key Laboratory of Quality Research in Chinese Medicine, Macau University of Science and Technology, Avenida Wai Long, Taipa, Macau, China; ^2^ Shum Yiu Foon Shum Bik Chuen Memorial Centre for Cancer and Inflammation Research, School of Chinese Medicine, Hong Kong Baptist University, Kowloon Tong, Hong Kong, China

**Keywords:** cysteine mutation, dihydromyricetin, IKK-β inhibitor, inflammation, NF-κB

## Abstract

Activation of IκB kinase β (IKK-β) and nuclear factor (NF)-κB signaling contributes to cancer pathogenesis and inflammatory disease; therefore, the IKK-β−NF-κB signaling pathway is a potential therapeutic target. Current drug design strategies focus on blocking NF-κB signaling by binding to specific cysteine residues on IKK-β. However, mutations in IKK-β have been found in patients who may eventually develop drug resistance. For these patients, a new generation of IKK-β inhibitors are required to provide novel treatment options. We demonstrate *in vitro* that cysteine-46 (Cys-46) is an essential residue for IKK-β kinase activity. We then validate the role of Cys-46 in the pathogenesis of inflammation using delayed-type hypersensitivity (DTH) and an *IKK-β*^C46A^ transgenic mouse model. We show that a novel IKK-β inhibitor, dihydromyricetin (DMY), has anti-inflammatory effects on WT DTH mice but not *IKK-β*^C46A^ transgenic mice. These findings reveal the role of Cys-46 in the promotion of inflammatory responses, and suggest that Cys-46 is a novel drug-binding site for the inhibition of IKK-β.

## INTRODUCTION

Disease pathogenesis, drug response, drug resistance, and drug toxicity are all correlated with the genetic background of individual patients [1-3]; therefore, personalized therapies targeting specific genes or gene mutations are highly desirable [4, 5]. Activation of nuclear factor-κB (NF-κB) transcription factors is commonly involved in the pathogenesis of immune diseases, inflammation, and cancer [6]. NF-κB activation is stimulated by a kinase complex, IκB kinase (IKK), which is composed of three core proteins: two catalytic subunits, IKK-α, IKK-β, and one regulatory subunit, IKK-γ (NEMO) [7]. Among these three subunits, IKK-β promotes NF-κB signaling and the pathogenesis and progression of inflammatory diseases [8]. There is also a causal link between hepatic IKK-β and NF-κB-accelerated sub-acute inflammation that culminates in chronic disease, such as obesity-induced insulin resistance [9, 10].

Suppression of the IKK-β-NF-κB signaling pathway has become a trendy new strategy for drug design. For example, several IKK-β inhibitors, such as beta-carbolines, BMS-345541, SC-514, CHS 828, and SAR113945, are currently in preclinical and clinical trials [11–19]. Each drugs' inhibitory activity varies depending on its binding affinity to specific functional sites on IKK-β, including the cysteine (Cys)-179 residue, the ATP binding domain, the allosteric domain, and serine (Ser)-177/-181 residues [12, 14, 15, 20, 21]. However, many of these sites, or other unidentified sites are variants in patients [7, 22–25], altering the effects of these drugs. Therefore, it is important to develop IKK-β inhibitors with new binding sites and/or tailor-make treatments for patients with IKK-β mutations. As cysteine residues participate in the catalysis and activation of IKK-β [26–28], the identification and characterization of cysteine mutations in IKK-β is essential to unravel the pathogenesis of IKK-β-related diseases and broaden the spectrum of novel IKK-β-based drug design [29–32].

In this study, we generated a new *IKK-*β*^C46A^* transgenic mouse model with a Cys-46 mutation in IKK-β, as well as a panel of IKK-β mutant constructs to investigate the role of the Cys-46 residue in the inflammatory process. We demonstrated that homozygosity for *IKK-*β*^C46A^* increases IKK-β kinase activity both *in vitro* and *in vivo*. We further showed that delayed-type hypersensitivity (DTH) in the homozygous *IKK-*β*^C46A^* mutant mice resulted in severe inflammation and diminished the anti-inflammatory effects of dihydromyricetin (DMY), a novel IKK-β inhibitor derived from the medicinal plant *Ampelopsis megalophylla*. These results suggest that Cys-46 is an important residue for the suppression of IKK-β kinase activity and inflammatory responses. Moreover, IKK-β Cys-46 mutant constructs and homozygous *IKK-*β*^C46A^* transgenic mice may be useful tools for drug screening and *in vivo* validation.

## RESULTS

### The small molecule dihydromyricetin (DMY) binds to Cys-46 of IKK-β and suppresses inflammation

**Figure 1:**
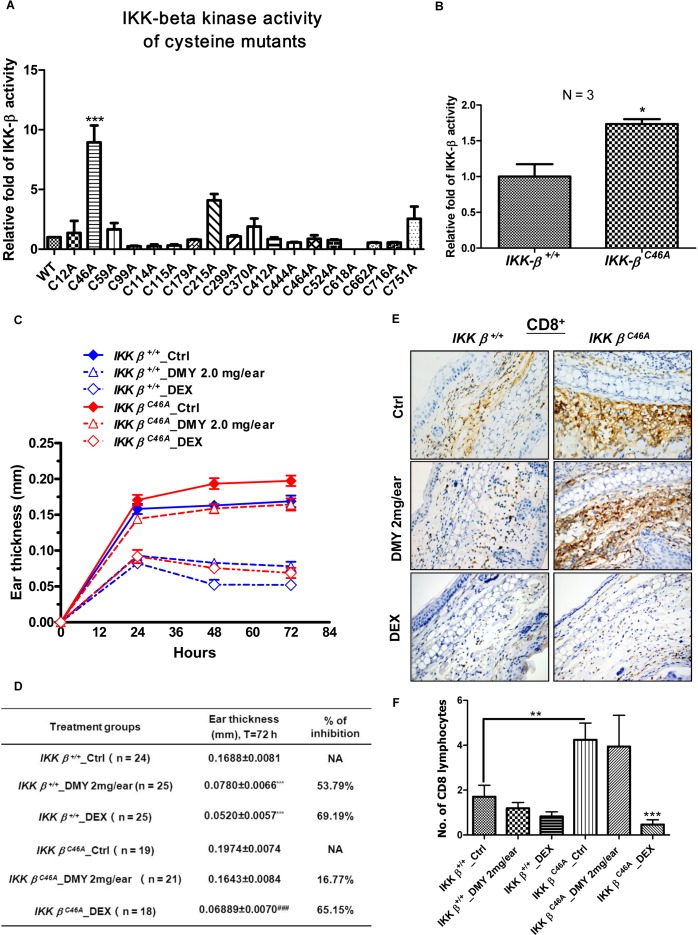
Homozygous IKK-β^C46A^ transgenic mice have a severe inflammatory response and are resistant to the IKK-β inhibitor DMY **A.** C46A mutation of IKK-β increased protein kinase activity *in vitro*. Human HEK293 cells transfected with WT or single-mutant FLAG-IKK-β plasmid were immunoprecipitated (IP) with anti-FLAG antibody, and the IP Flag-IKK-β was incubated with GST-IκBα substrate and ATP for 2 h. The IKK-β kinase activity was determined by the level of phosphorylated GST-IκBα using antibody against p-IκBα. **B.** C46A mutation of IKK-β increased protein kinase activity *in vivo*. Protein lysates extracted from kidney tissues of both *IKK-*β WT and *IKK-*β*^C46A^* transgenic mice (*N* = 3 for each group) were IP with anti-IKK-β antibody, then subjected to an *in vitro* IKK-β kinase assay using GST-IκBα substrate. The bar chart shows relative WT and mutant IKK-β kinase activity. **C.** DTH immunological study using homozygous *IKK-*β*^C46A^* mutant mice. *IKK-*β WT and *IKK-*β*^C46A^* mice challenged with DNFB (left ear only) were treated with DMY (2.0 mg per ear) or dexamethasone (0.025 mg per ear) for 72 h. Ear swelling and thickness were measured in millimeters. Each measurement represents the mean ± SEM of the increase in ear swelling in the left ear compared to the right ear of the same animal. *P < 0.05, **P < 0.01 by Dunnett's multiple comparison test. **D.** Inflammatory responses and resistance to the small-molecule IKK-β inhibitor DMY in the DTH assay in *IKK-*β WT and *IKK-*β*^C46A^* transgenic mice. **E.** Immunohistochemical analysis of CD8^+^ T lymphocytes in the ear tissues of DTH*-IKK-*βWT and *-IKK-*β*^C46A^* mice. **F.** The average number of CD8^+^ T lymphocytes found in the ear sections of WT and mutant DTH animals.

Using site-directed mutagenesis, we found that mutation of IKK-β cysteine-46 to alanine (C46A) increased kinase activity *in vitro* (Figure 1A). To assess the function of this mutant kinase *in vivo*, we generated homozygous *IKK-*β*^C46A^* transgenic (*IKK-*β*^C46A^*) mice and examined their inflammatory response to dinitrofluorobenzene (DNFB). When compared with wild-type (WT) mice, IKK-β protein immunoprecipitated from *IKK-*β*^C46A^* kidneys had increased kinase activity (Figure 1B). *IKK-*β*^C46A^* mice treated with DNFB displayed stronger inflammatory responses than WT mice, with increased ear thickness (Figure 1C & 1D). Taken together, these results indicate that cysteine-46 is a reactive residue that regulates IKK-β kinase activity.

Given that reactive cysteines can bind with small molecules via redox reactions or Michael addition [28], we next examined whether the small molecule, dihydromyricetin (DMY), could bind with cysteine-46 to exert an anti-inflammatory effect. DMY suppressed IKK-β-NF-κB signaling, T cell activation, and cytokine production in purified human T lymphocytes (Figure S1 & S2), but its anti-inflammatory effects were diminished in *IKK-*β*^C46A^* mice (Figure 1C & 1D). DMY treatment (2 mg/ear) caused a 53.79% suppression of DNFB-mediated ear edema in WT mice, whereas this suppression was only 16.77% in *IKK-*β*^C46A^* mice (Figure 1D). By contrast, dexamethasone (DEX), showed similar suppressive effects in both WT and *IKK-*β*^C46A^* mice (Figure 1C & 1D). These results suggest that *IKK-*β*^C46A^* are resistant to DMY treatment.

Effector CD4^+^ and CD8^+^ lymphocytes are stimulated in DNFB-induced DTH [33], and are increased in ear sections of DNFB-treated *IKK-*β*^C46A^* mice when compared to WT. While the number of CD8^+^ lymphocytes gradually decreases in WT mice, this does not occur in *IKK-*β*^C46A^* mice (Figure 1E &1F& Figure S3), suggesting that CD8^+^ lymphocytes are involved in the anti-inflammatory actions of DMY [4].

**Figure 2:**
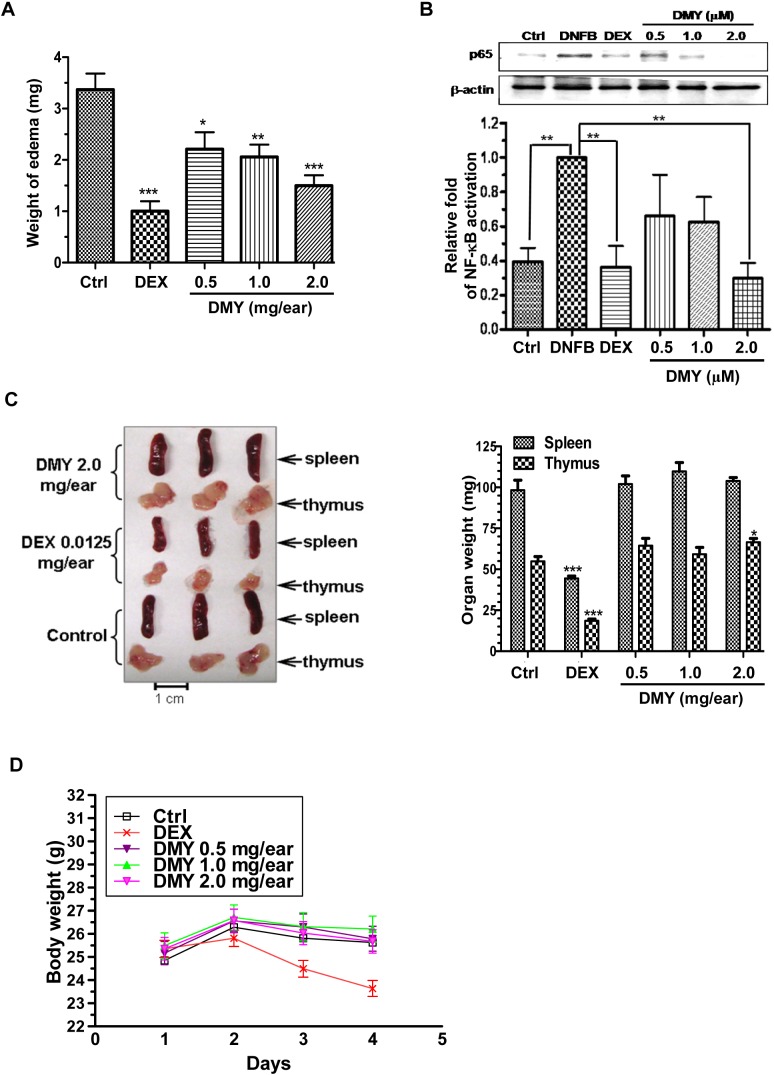
Anti-inflammatory effects of DMY in DNFB-induced DTH mice **A.** DMY dose-dependently inhibited acute ear edema of DTH-mice. **B.** DMY suppressed the nuclear translocation of NF-κB p65 in the ear tissues of DTH-mice. The bar chart shows the quantitation of Western blot results from three different animals within the same treatment groups. **C.** & **D.** DMY treatment did not affect the weight of the spleen, thymus or whole body in DTH-mice. Five groups of mice were challenged with DNFB to develop DTH. The DTH mice were then treated with or without the reference drug DEX or the indicated dosage of DMY for 4 days. The acute ear edema tissue, spleen and thymus of DTH mice were dissected and weighed at the end of drug treatment, while the body weight of animals was assessed daily. The nuclear protein lysates of the ear tissues of DTH mice were prepared for Western blotting analysis using the antibody against p65. Data are expressed as means ± SEM (n = 9-12), *P < 0.05, **P < 0.01, ***P < 0.001 compared to vehicle-treated mice.

**Figure 3:**
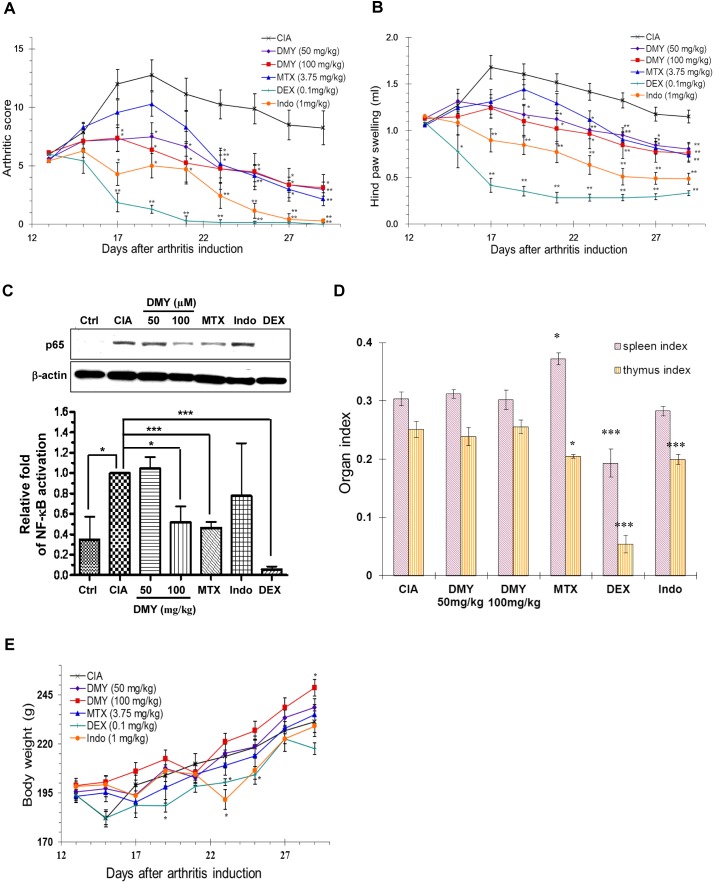
Anti-arthritic effect of DMY in collagen-II induced arthritis (CIA) rats **A.** DMY dose-dependently reduced the arthritic score of CIA rats. **B.** DMY dose-dependently ameliorated the hind paw swelling of CIA rats. **C.** DMY suppressed the nuclear translocation of NF-κB p65 in the knee synovial tissues of CIA rat. The bar chart represents the quantitation of Western blots resulting from three different animals within the same treatment groups. **D.** DMY did not impair the organ indexes of CIA rats. **E.** Effect of DMY on the body weight change of CIA rats. Six groups of rats were treated daily with DMY at 50 (♦) and 100 mg/kg (■), MTX at 3.75 mg/kg (▲), DEX at 0.1 mg/kg (+), indomethacin at 1 mg/kg (●) or vehicle (x) from day 13 after arthritis induction until day 30. All of the hind paw volumes (mL), arthritic scores and body weights were determined daily, while the organ indexes (spleen or thymus weight over body weight) were calculated for each CIA rat at the end of the experiment. The nuclear protein lysates from the knee synovial tissues of DMY-treated rats were prepared for Western blotting analysis using the antibody against p65. Data are expressed as means ± SEM (*n* = 7-8). *p < 0.05, ** *p* < 0.01 compared to vehicle-treated CIA rats.

Topical application of DMY reduced ear edema in a dose-dependent manner (Figure 2A) by suppressing p65 NF-κB signaling in ear tissues of the DMY-treated DTH mice (Figure 2B). DMY treatment caused no adverse effects to spleen or thymus and no loss of body weight (Figure 2C & 2D), while adverse responses were observed in DEX-treated mice. In the Collagen Induced Arthritis (CIA) rat model [12], DMY reduced arthritic scores and hind paw volume in comparison with vehicle-treated CIA rats (Figure 3A & 3B). DMY also suppressed p65 NF-κB signaling in knee synovial tissues of the CIA rats (Figure 3C), without impairment to the organ indexes (Figure 3D) or body weights (Figure 3E). Taken together, our data suggest that DMY binds to Cys-46 of IKK-β and suppresses inflammation *in vivo*.

### DMY covalently binds the Cys-46 binding site of IKK-β

**Figure 4:**
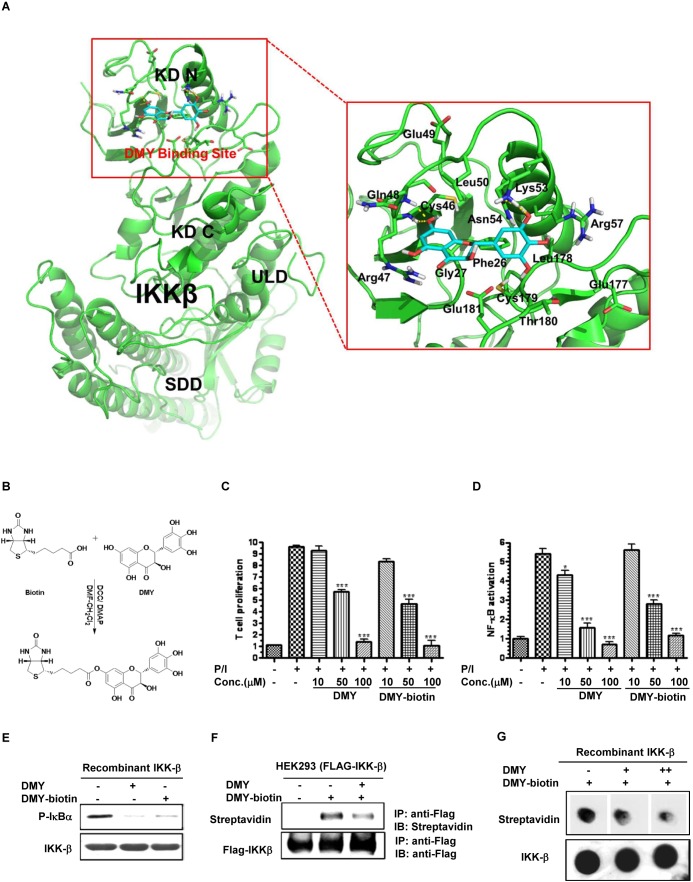
Determination of the DMY-binding region on IKK-β via computational docking analysis and biotinylated probes **A.** The binding mode of DMY on IKK-β. IKK-β protein was represented as a cartoon. DMY and key residues around the binding pocket were shown as sticks. The hydrogen bond is represented by a yellow dashed line. Hydrophobic interactions were predicted between DMY and the side chain of Phe-26, Gly-27, Cys-46, Leu-50, Leu-178, Cys-179, and Thr-180. Polar interactions were predicted between DMY and the side chain of Arg-47, Gln-48, Asn-54 and Glu-181. DMY was predicted to form hydrogen bonds with the backbone of Cys-46 and Gln-48 and the side chain of Lys-53 and Asn-54. **B.** Biotinylated DMY (DMY-biotin) was synthesized as a probe to determine the binding site of DMY. **C.** DMY-biotin exhibited a similar suppression effect as DMY on T cell proliferation. Cell proliferation was calculated as the relative amount of BrdU incorporation over the untreated controlled cells. **D.** DMY-biotin exhibited a similar inhibitory effect as DMY on NF-κB transcriptional activity. Cellular lysates from NF-κB luciferase reporter-transfected Jurkat cells were used to determine the NF-κB transcriptional activity using a Luciferase Reporter Assay System (Promega). Values represent means ± SEM (*n* = 3, *P < 0.05, ***P < 0.001, compared with untreated controlled cells). **E.** DMY-biotin directly suppressed IKK-β kinase activity, like DMY. His-tagged human recombinant IKK-β (2 ng) was incubated with GST-IκBα substrate and 10 mM ATP in the presence of 100 μM of DMY or DMY-biotin. (F & G) DMY-biotin competed with the same binding region of DMY on IKK-β protein. His-tagged human recombinant IKK-β (2 ng) was incubated with 100 μM DMY-biotin in the presence of 100 μM (+) or 200 μM (++) unlabeled DMY compound. The DMY-biotin signal was visualized by streptavidin peroxidase.

Computational docking analysis gave a docking score of −14.06 to DMY with the binding domain of IKK-β, but their binding mode was not facilitated by hydrophobic, polar, or hydrogen-bond interactions. Accordingly, DMY was predicted to form hydrogen bonds with the backbone of Cys-46, Gln-48, Lys-53 and Asn-54 in the proposed IKK-β binding domain (Figure 4A), indicating that Cys-46 might be a key residue to facilitate DMY binding. To determine the nature of the interaction between DMY and Cys-46 of IKK-β and detect their direct binding, biotinylated DMY (DMY-biotin) was synthesized as a probe (Figure 4B). Functional assays showed that DMY-biotin suppressed PMA/ionomycin (P/I)-activated T cell proliferation, NF-κB signaling, and IKK-β kinase activity similarly to normal DMY (Figure 4C–4E), indicating that addition of the biotin moiety did not impair DMY function. A competitive binding assay revealed direct binding between DMY-biotin and IKK-β, showing clearly formed DMY-biotin/FLAG-tagged IKK-β adducts, whereas addition of unlabeled DMY reduced this binding (Figure 4F). Furthermore, binding of DMY-biotin to recombinant IKK-β was dose-dependently competed away by unlabeled DMY (Figure 4G), suggesting that the binding sites of DMY and DMY-biotin are conserved. Although our computational docking predicted only the hydrogen-bond interaction between DMY and Cys-46 of IKK-β, the DMY-biotin/FLAG-tagged IKK-β adducts detected by SDS-PAGE suggested the covalent linkage of DMY with IKK-β protein (Figure 4F).

### DMY suppresses IKK-β activity via a novel drug binding site

**Figure 5:**
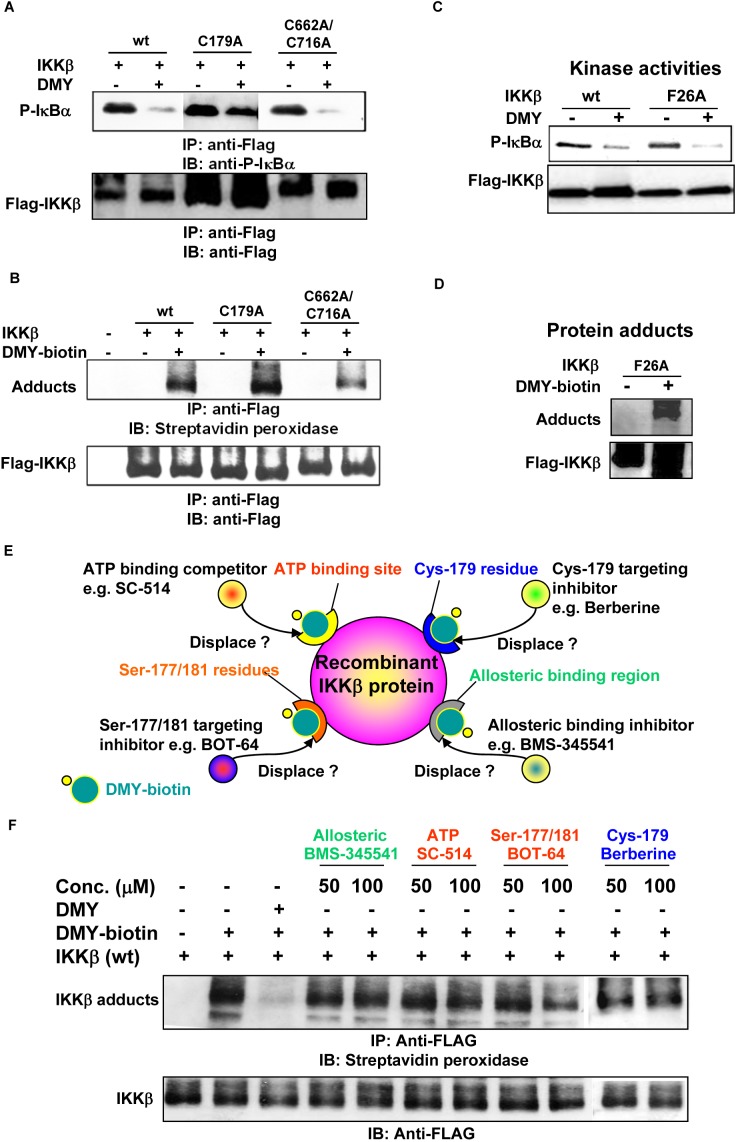
Identification of a novel drug-binding site on IKK-β using the small molecule DMY **A.** DMY inhibited the kinase activity of IKK-β (C179A) and IKK-β (C662A/C716A). **B.** DMY-biotin formed protein adducts with IKK-β (C179A) and IKK-β (C662A/C716A). **C.** DMY circumvented the drug-resistant phenotype of IKK-β (F26A) with an ATP-binding site mutation. **D.** DMY-biotin formed protein adducts with IKK-β (F26A). HEK293 cells transfected with WT or mutant FLAG-IKK-β plasmid were immunoprecipitated for kinase activity assay and protein-adduct formation assay. The protein adducts formed between mutant IKK-β and DMY-biotin were visualized by streptavidin peroxidase. **E.** Schematic diagram showing the novel site on IKK-β responsible for DMY binding. **F.** DMY-biotin probe assisted IKK-β displacement binding assay. HEK293 cells transfected with FLAG-IKK-β(WT) plasmid were immunoprecipitated (IP) with anti-FLAG antibody. The IP Flag-IKK-β was incubated with 100 μM DMY-biotin in the presence of the indicated concentrations of DMY, berberine, SC-514, BMS-345541 or BOT-64. The DMY-biotin signal was detected by Western blotting using streptavidin peroxidase.

We next characterized the binding capability of DMY with the known drug-targeted regions of IKK-β, including the Cys-179 binding site for manumycin A, butein, berberine and arsenite [21, 26, 27, 34], and the homotypic dimerization residues Cys-662/-716 for manumycin A [26]. Using an *in vitro* IKK-β kinase assay, we found that DMY suppressed IκBα phosphorylation in both WT and mutant (C179A and C662A/C716A) IKK-β transfectants (Figure 5A). In addition, incubation of both WT and mutant IKK-β proteins with DMY-biotin led to the formation of IKK-β-DMY adducts (Figure 5B). Thus, Cys-179, -662 and -716 on IKK-β are likely not the binding sites of DMY. Other than these cysteine residues, Phe-26 (F26) in the ATP-binding site of IKK-β is the key residue for the ATP-binding competitor PHA-408 [35]. DMY effectively suppressed the kinase activity of an F26A IKK-β mutant compared to the IKK-β WT (Figure 5C), and the labeled DMY probe bound and formed protein adducts with this mutant (Figure 5D), indicating that DMY does not bind to Phe-26. Next, we used DMY-biotin in an IKK-β displacement assay to compete with well-known IKK-β inhibitors: SC-514, an ATP-competitive inhibitor; berberine, a Cys-179-targeting inhibitor; BMS-345541, an allosteric domain inhibitor; and BOT-64, a Ser-177/-181-targeting inhibitor (Figure 5E). None of these inhibitors competed with DMY-biotin (Figure 5F). Thus, DMY suppresses kinase activity and decreases inflammation via a novel drug-binding region on IKK-β.

### The Cysteine-46 residue, a novel DMY binding site on IKK-β is responsible for suppression of IKK--β − ΝF-κB signaling

**Figure 6:**
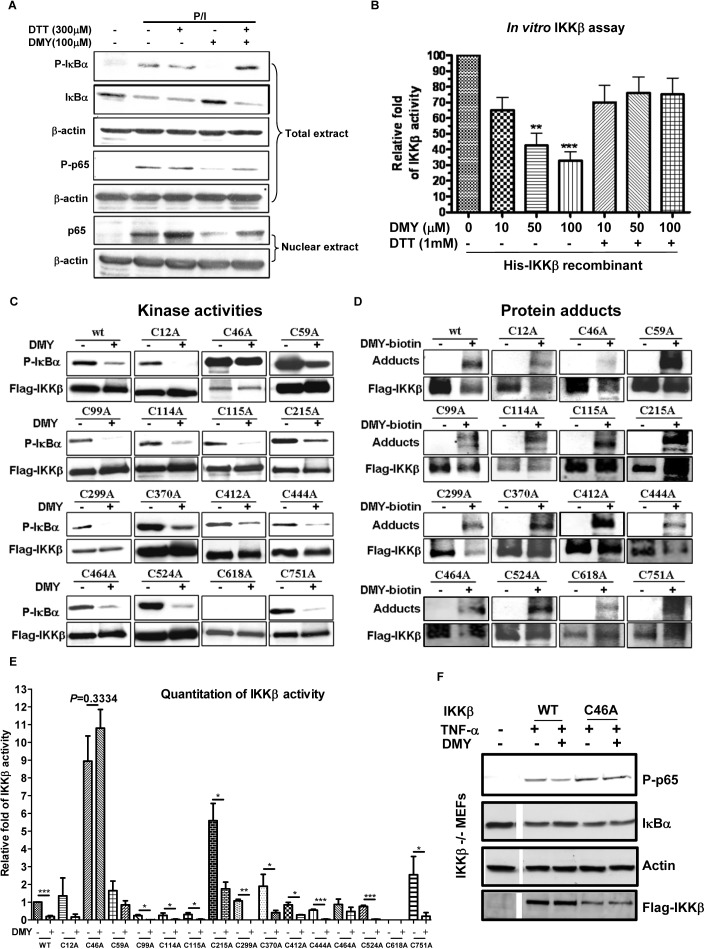
Validation of Cys-46 as a novel drug-binding site on IKK-μ using DMY **A.** Inhibitory effects of DMY on P/I-induced activation of NF-κB signaling was blocked by dithiothreitol (DTT). Isolated human T lymphocytes were pre-treated with 300 μM DTT and 100 μM DMY followed by P/I stimulation. Total cell extracts and nuclear extracts were then prepared for Western blotting analysis using the antibodies against p-IκBα, IκBα, p-p65, p65 and β-actin. **B.** The inhibitory effect of DMY on IKK-β kinase activity was reversed by DTT. His-tagged human recombinant IKK-β (2 ng) was incubated with GST-IκBα substrate, 10 mM ATP, and the indicated concentrations of DMY in the absence or presence of 1 mM DTT. Kinase activity was measured by spectrophotometer at O.D. 450 nm and the results were calculated as relative IKK-β activity over untreated control. **P < 0.01, ***P < 0.001, compared with untreated control. **C.** Mutation of IKK-β Cys-46 abrogated the kinase-inhibitory effect of DMY. **D.** Mutation of IKK-β Cys-46 abrogated the formation of protein adducts with DMY. HEK293 cells transfected with WT FLAG-IKK-β or mutant plasmids with single point mutations on cysteine residues were immunoprecipitated (IP) with anti-FLAG antibody, and the IP Flag-IKK-β was incubated with GST-IκBα, substrate and ATP in the presence or absence of 100 μM DMY. The IKK-β kinase activity was determined by the level of phosphorylated GST-IκBα using antibody against p-IκBα. For detection of protein adducts formed between DMY-biotin and Flag-IKK-β protein, the DMY-biotin signal was analyzed by Western blot using streptavidin peroxidase. **E.** Bar chart represented the Western blot quantitation of mutant IKK-β kinase activity. **F.** The Cys-46 residue was crucial for the DMY-mediated suppression of IKK-β-NF-κB signaling *in vitro. IKK-*β*^−/−^* MEFs transfected with WT or C46A FLAG-IKK-β were pre-treated with or without 50 μM DMY, followed by 20 ng/mL of TNF-α, then subjected to Western blotting using antibodies against phospho-p65 and IκBα.

The thiol-containing cysteine residue of IKK-β is bound and suppressed by cyclopentenone prostaglandins and CDDO, and these interactions are abolished by dithiothreitol (DTT) [28, 29]. DMY-induced suppression of IKK-β-NF-κB signaling was reversed by DTT, as well (Figure 6A & 6B), suggesting that DMY suppresses IKK-β activity via the thiol-containing cysteine residues of IKK-β. To further determine whether the cysteine residue(s) on IKK-β are essential for the anti-inflammatory actions of DMY, we used site-directed mutagenesis to generate all 15 remaining IKK-β point mutants by replacing cysteine (C) with alanine (A) (Figure 6C). Except C618A, all the mutants retained their kinase activities, as determined by phosphorylation of IκBα. Importantly, mutation of Cys-46 (C46A) abrogated the inhibitory effects of DMY, on IKK-β kinase activity and the formation of protein adducts with DMY-biotin (Figure 6C, 6D, 6E). We next derived embryonic fibroblasts (MEFs) from IKK-β-deficient (*IKK-*β*^−/−^*) mice. *IKK-*β*^−/−^* MEFs transfected with WT IKK-β, but not with IKK-β (C46A) displayed reduced TNF-α-induced phosphorylation of NF-κB p65 and degradation of IκBα (Figure 6F), further confirming that Cys-46 on IKK-β is the key residue for DMY-induced suppression of NF-κB signaling.

## DISCUSSION

Somatic mutations in target genes are major impediments to successful targeted cancer therapies. For instance, the survival rates of non-small-cell lung cancer (NSCLCs) patients with EGFR mutations were improved by first-generation epidermal growth factor receptor (EGFR) kinase inhibitors, gefitinib and erlotinib; however, 50% of these patients will acquire resistance and ultimately relapse due to a second mutation on EGFR (T790M) [36, 37]. In 2009, WZ4002, a new mutant-selective EGFR kinase inhibitor was identified which selectively inhibits EGFR in patients with the T790M mutation [38].

Nonsense and frameshift mutations of IKK-β have been identified in patients who present with a severe combined immunodeficiency (SCID) disease phenotype [7, 22–25, 39–41]. Moreover, mutations in IKK-β and its related genes, such as IκBα, IKK-β and IKK-γ are commonly found in patients with immune deficiencies and inflammatory diseases [7], suggesting that somatic mutations often occur among patients with inflammatory conditions. Among the known drug-binding regions of IKK-β, mutations of Phe-26 at the ATP binding site or Cys-179 in the kinase domain abrogate the kinase-inhibiting effects of PHA-408 [35] and other Cys-179-targeting compounds [21, 26, 27, 29, 34, 42, 43]. Patients suffering from inflammatory and autoimmune diseases are usually treated with drugs possessing IKK-β inhibitory effects, such as the ATP-competitive IKK-β inhibitor, sulfasalazin [44] or Cys-179-targeting compounds including xanthohumol [45] and TPCK [46]. Patients with long-term treatment will develop drug resistance due to IKK-β somatic mutations in drug binding sites [47–50]. Therefore, to overcome drug-resistant phenotypes in immune diseases caused by somatic mutations, we need to discover novel IKK-β inhibitors with different inhibitory mechanisms or new drug binding domains.

In our study, we found a new inflammation-promoting role for Cys-46 in IKK-β. We demonstrated that DMY has an inhibitory effect on IKK-β kinase activity and suppresses NF-κB signaling through direct binding to the novel Cys-46 drug binding site (Australia Innovation Patent: 2013101130). Recently, a natural compound, ainsliadimer A, was found to selectively bind to the Cys-46 residue of IKK-α/β and inhibit its activities through an allosteric effect [51]. Our data is in line with their data, and further supports that Cys-46 residue is a new drug-binding site for kinase inhibition. Importantly, DMY was fully validated both by *in vitro* and *in vivo* models as a drug that can overcome the drug-resistant phenotypes of Cys-179 (C179A) and ATP binding site (F26A) mutants of IKK-β. Once FDA-approved IKK-β inhibitors have been given to patients, drug-resistant patients with IKK-β mutant genotypes could eventually be treated with the IKK-β inhibitor, DMY.

## MATERIALS AND METHODS

### Chemicals, antibodies, plasmids and animals

Dihydromyricetin (DMY) (purity > 98%, verified by HPLC-DAD and LC-MS analysis) was obtained from Ze Lang Biotechnology Company Ltd. (Nanjing, China). SC-514, BOT-64 and BMS-345541 were purchased from Calbiochem (San Diego, CA, USA). PE- or FITC-labelled antibodies against CD3, CD25, CD69, CD71, CD28 and OKT3 were from BD Pharmingen Inc. (San Diego, CA, USA). Phorbol 12-myristate 13-acetate (PMA) and ionomycin were obtained from Sigma and Calbiochem, respectively. All primary antibodies used in this study were purchased from Santa Cruz, Calbiochem, Cell Signaling, BD Pharmingen or Sigma. The expression plasmids of FLAG-tagged IKKβ and its single mutant C179A and the double mutant C662A/C716A were gifts from Professor Tom Gilmore (Boston University). DMY was encapsulated with hydroxypropyl cyclodextrin (HP-CD) (1:8.48) and then dissolved in normal saline for administration at dosages of 50 and 100 mg/kg/day. To measure the acute toxicity of DMY, the average LD_50_ of DMY was 1251.11 mg/kg/day in mice by intraperitoneal injection. Male ICR mice weighing 22-30 g and female Wistar rats that were 5-6 weeks old were obtained from the Laboratory Animal Services Center, Chinese University of Hong Kong (Hong Kong, China). All animals were fed a standard diet *ad libitum*. Housing conditions and all *in vivo* experiments were approved under regulations of the Committee on Use of Human and Animal Subjects in Teaching and Research (HASC) of the Department of Health in Hong Kong.

### Generation of IKK-β*^C46A^* transgenic mice

*IKK-*β*^C46A^* transgenic mice were generated by Shanghai Biomodel Organism Science & Technology Development Co., Ltd. In brief, an *IKK-*β*^C46A^* targeting construct with a Neo resistance gene was linearized and transfected into ES cells (129 mouse strain, SCR012, Chemicon) by electroporation. The selected recombinant ES cells were then injected into blastocysts to generate *IKK-*β*^C46A^* transgenic mice. The knockout clones were validated by PCR and gene sequencing using the following primers: Forward primer: GTGATGTGGGGGTGGTGAGG; Reverse primer: TTTGGGCTGAGCTCCTGTCG. The PCR conditions were denaturing at 94°C for 5 min, 35 cycles of denaturing at 94°C for 30 sec, annealing at 66°C for 30 sec and elongation at 72°C for 30 sec, and a final elongation at 72°C for 10 min.

### Delayed-type hypersensitivity test and immunohistochemical staining

The *in vivo* anti-inflammatory effect of DMY was examined by using the mouse delayed-type hypersensitivity (DTH) test based on a previously described method [52]. The ear samples of DTH mice were fixed in 4% neutral-buffered formalin. Each sample was cut longitudinally in half and embedded in paraffin (Panreac), and then cut into 5 μm sections. Immunohistochemical staining was performed on slides according to a previous report [53]. In brief, sections were deparaffinized in xylene, subsequently immersed in graded alcohol (100%, 95%, 70% ethanol), rehydrated in ddH_2_O before antigen retrieval by microwave. The sections were incubated with antibodies against CD4 (1:20 dilution) and CD8 (1:100 dilution) (BD Pharmingen Inc.) at 4°C overnight. The LSAB^TM+^ system (Dako Com., CA) was used to detect these antigens in tissues. The sections were developed with DAB and lightly counterstained with hematoxylin, then rinsed and immersed briefly in acid alcohol. The slides were further rinsed and soaked in Scott's tap water for 5 min. Finally, slides were dehydrated and mounted with Permount and covered with Fisherfinest glass cover slips (Thermo-Fisher) for microscopic observation and scoring.

### Experimental arthritis induced by collagen II in rats

Collagen II-induced arthritis (CIA) was induced in female Wistar rats as described previously [54]. Dexamethasone (DEX, 0.1 mg/kg), methotrexate (MTX, 3.75 mg/kg, twice per week) and indomethacin (Indo, 1 mg/kg) were used as reference drugs.

### Computational docking

The initial 3D structure of DMY was built using the Molecule Builder module incorporated in MOE software. The crystal structure of IKK-β was retrieved from Protein Data Bank (PDB ID code 3RZF [55]. The DMY structure was then subjected to energy minimization and partial charges calculation with an Amber99 force field. Energy minimization was terminated when the root mean square gradient fell below 0.05 kcal/(mol·Å). The docking sites were identified using Site Finder in MOE software. The identified binding sites, including residue Cys-46, were chosen as the binding sites for molecular docking according to our experiment. In molecular docking, the Triangle Matcher placement method and London dG scoring function were used. A total of 30 docking poses were generated for the ligand, and the pose with the best binding mode was selected for further analysis.

### IKK-β kinase assay and NF-κB luciferase reporter assay

An IKK-β kinase assay was performed using a K-LISA^TM^ IKKβ-Inhibitor Screening Kit (Calbiochem) as described previously [56].

### Synthesis of biotinylated DMY (DMY-Biotin)

Biotin (24.4 mg, 0.1 mmol) was suspended in dimethylformamide/dichloromethane (1:1, 2 mL), and dicyclohexylcarbodiimide (20.6 mg, 0.1 mmol) was added. After stirring at 60°C for 5 min, dimethylaminopyridine (12.2 mg, 0.1 mmol) and DMY (48 mg, 0.15 mmol) in dimethylformamide (0.5 mL) were added. After stirring overnight, the mixture was poured into water (50 mL), acidified with 3 M HCl to pH 3.0, and then extracted with ethyl acetate (20 mL ×3). Residue of the organic layer was subjected to silica gel chromatography (petroleum ether: acetone from 4:3 to 1:3) to produce a yellow solid (25.1 mg, 46%). Negative HR-ESI-MS with *m/z* 545.1203 [M-H]^−^ was determined from biotinylated DMY (calculated for C_25_H_25_N_2_O_10_S: 545.1230).

### Binding of DMY-biotin to IKKβ

HEK293 cells were transfected with a WT or mutant FLAG-IKKβ construct using Lipofectamine PLUS LTX reagent (Invitrogen) according to the manufacturer's instructions. Whole-cell lysates were then immunoprecipitated with anti-FLAG antibody and then incubated with 100 μM DMY-biotin. The immunoprecipitated FLAG-IKK-β-DMY-biotin ligands were separated by SDS-PAGE and transferred onto nitrocellulose membranes. After blocking with 1% BSA and washing with PBS-T [Tween-20 0.05%], the membranes were incubated with streptavidin horseradish peroxidase (Sigma) for 1 h and developed using ECL Western Blotting Detection Reagents (Invitrogen).

### IKK-β competition and displacement assay

For the IKK-β competition assay, recombinant IKK-β was incubated with 100 μM DMY-biotin with addition of 100 μM or 200 μM unlabeled DMY at room temperature for 1 h. The mixtures were then pipetted onto nitrocellulose membrane, followed by BSA blocking and PBS-T washing. For the IKK-β displacement assay, FLAG-IKK-β (wt)-transfected human HEK293 cells were lysed and immunoprecipitated with anti-FLAG antibody, then incubated with 100 μM DMY-biotin in the presence of DMY, berberine, SC-514, BMS-345541 or BOT-64. The reaction samples were separated by SDS-PAGE and analyzed by Western blotting using streptavidin horseradish peroxidase antibodies.

### Cloning and expression

The FLAG-IKK-β (wt) construct was used as a template to introduce the point mutations of cysteine (C) or phenylalanine (F) replaced by alanine (A), including C12A, C46A, C59A, C99A, C114A, C115A, C215A, C299A, C370A, C412A, C444A, C464A, C524A, C618A, C751A and F26A mutations. Site-directed mutagenesis was performed using the Stratagene QuickChange Mutagenesis Kit according to the manufacturer's instructions. *IKK-*β*^−/−^* mouse embryonic fibroblasts (MEFs) were provided as a gift by Prof Michael Karin (University of California, San Diego).

### Human T lymphocyte isolation, purification, stimulation and proliferation

Human peripheral blood T lymphocytes were isolated from buffy coat blood and were activated following previous stimulation protocols [57, 58]. Briefly, the buffy coat provided by Macao Blood Transfusion Centre or Red Cross Association of Hong Kong was mixed with normal saline, and then added to 50 mL centrifuge tube containing Ficoll-Pague plus (Amersham Biosciences, USA). After centrifugation at 350 g for 35 min, the mixture was separated into several layers. The layer of mononuclear cells was collected and purified by magnetic-activated cell sorting (MACs) pan T cell kit (Miltenyi Biotec, Germany). The purified T cells were stimulated by 20 ng/mL PMA plus 1 μM ionomycin (P/I) or OKT3 (5 μg/mL) plus CD28 mAb (1 μg/mL) in the experiments with different time intervals for later experiments. The effects of DMY on T cell proliferation were investigated by the cell proliferation kit (Roche, USA) according to the manufacturer's instruction. In brief, human T lymphocytes (1×10^5^/well) were cultured in 96-well plates in triplicate in RPMI 1640 media plus 10% FBS, and then stimulated with 20 ng/mL PMA plus 1 μM ionomycin or OKT3 (5 μg/mL) plus CD28 mAb in the presence or absence of DMY for 72 h. Before the cells were collected, BrdU was added for 14 h incubation at final concentration of 10 μM. Finally, BrdU levels were determined by ELISA according to kit instructions. Data were obtained from three independent experiments.

### Measurement of IL-2 and T lymphocyte surface markers

The level of IL-2 produced by activated human T lymphocytes was evaluated using an IL-2 ELISA (BD Pharmingen). The expression of T lymphocyte surface markers, CD25, CD69 and CD71, was determined by a BD FACSCanto flow cytometer using BD FACSDiva software as described previously [56].

### Statistical analysis

The data were analyzed by SPSS and Graphpad Prism. Results are expressed as the means ± SEM. Differences were considered statistically significant when the *P*-value was less than 0.05. Student's *t*-test or one-way ANOVA was used for comparison between groups.

## SUPPLEMENTARY MATERIAL FIGURES


