# Supplementary material for: Mutation of cysteine 46 in IKK-beta increases inflammatory responses
Source: Oncotarget. 2015 Sep 10;6(31):31805–19. doi: 10.18632/oncotarget.5567 (PMC4741641; doi:10.18632/oncotarget.5567)
Supplement: Supplementary file 1 [file oncotarget-06-31805-s001.pdf]

Mutation of Cysteine 46 in IKK-beta increases inflammatory responses

Supplementary Material

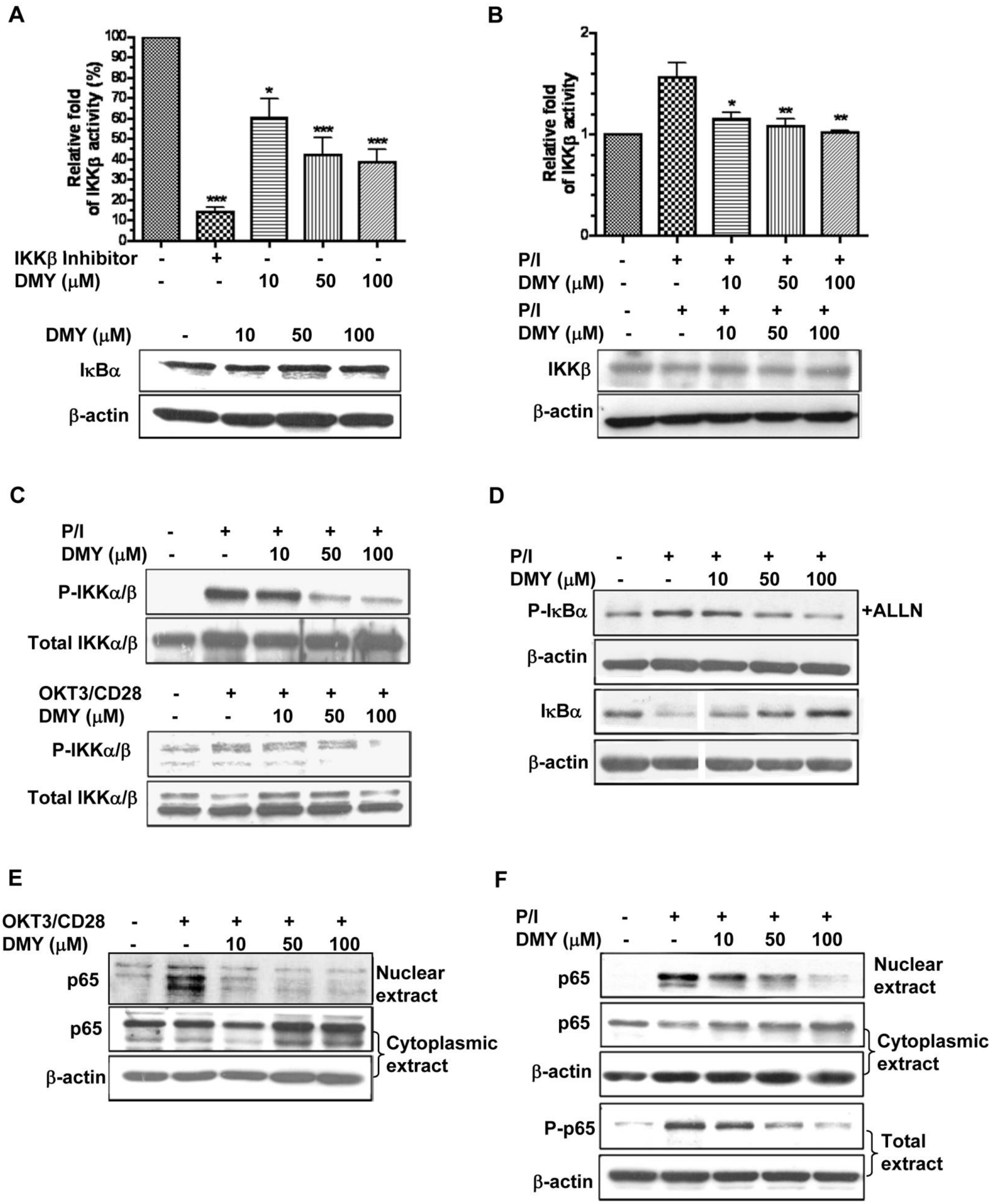

Fig. S1. Suppression of IKK-β–NF-κB signalling by DMY.

(A) Upper panel: DMY directly inhibited IKK-β activity. His-tagged human recombinant IKK-β (2 ng) was incubated with GST-IκBα substrate in the presence of the indicated concentrations of DMY and 10 mM ATP. IKK-β inhibitor was the positive control provided in the screening kit. Lower panel: the expression of IκBα in response to DMY treatment. (B) Upper panel: DMY suppressed PMA/ionomycin (P/I)-induced IKK-β activity in the isolated human T lymphocytes. Lower panel: the expression of IKK-β in response to DMY treatment. The isolated human T lymphocytes were treated with the indicated concentrations of DMY and then stimulated with 20 ng/ml PMA plus 1 μM ionomycin (P/I). Whole-cell extracts were harvested for IKK-β kinase assay or Western blotting. (C) DMY suppressed P/I- or OKT-3/CD28-mediated phosphorylation of IKK-α/β. (D) DMY prevented P/I-induced phosphorylation and degradation of IκBα. (E & F) DMY abrogated the OKT-3/CD28- or P/I-mediated phosphorylation and nuclear translocation of NF-κB p65. Isolated human T lymphocytes were pretreated with the indicated concentrations of DMY, followed by co-stimulation by P/I, or the cells were treated with DMY with or without OKT-3/CD28 antibodies. For detection of phosphorylation of IκBα, the cells were first incubated with DMY plus 100 μM ALLN prior to P/I stimulation. Data represented the mean ± SEM of three independent experiments. * $P<0.05$, **$P<0.01$, ***$P<0.001$, compared with P/I treatment alone.

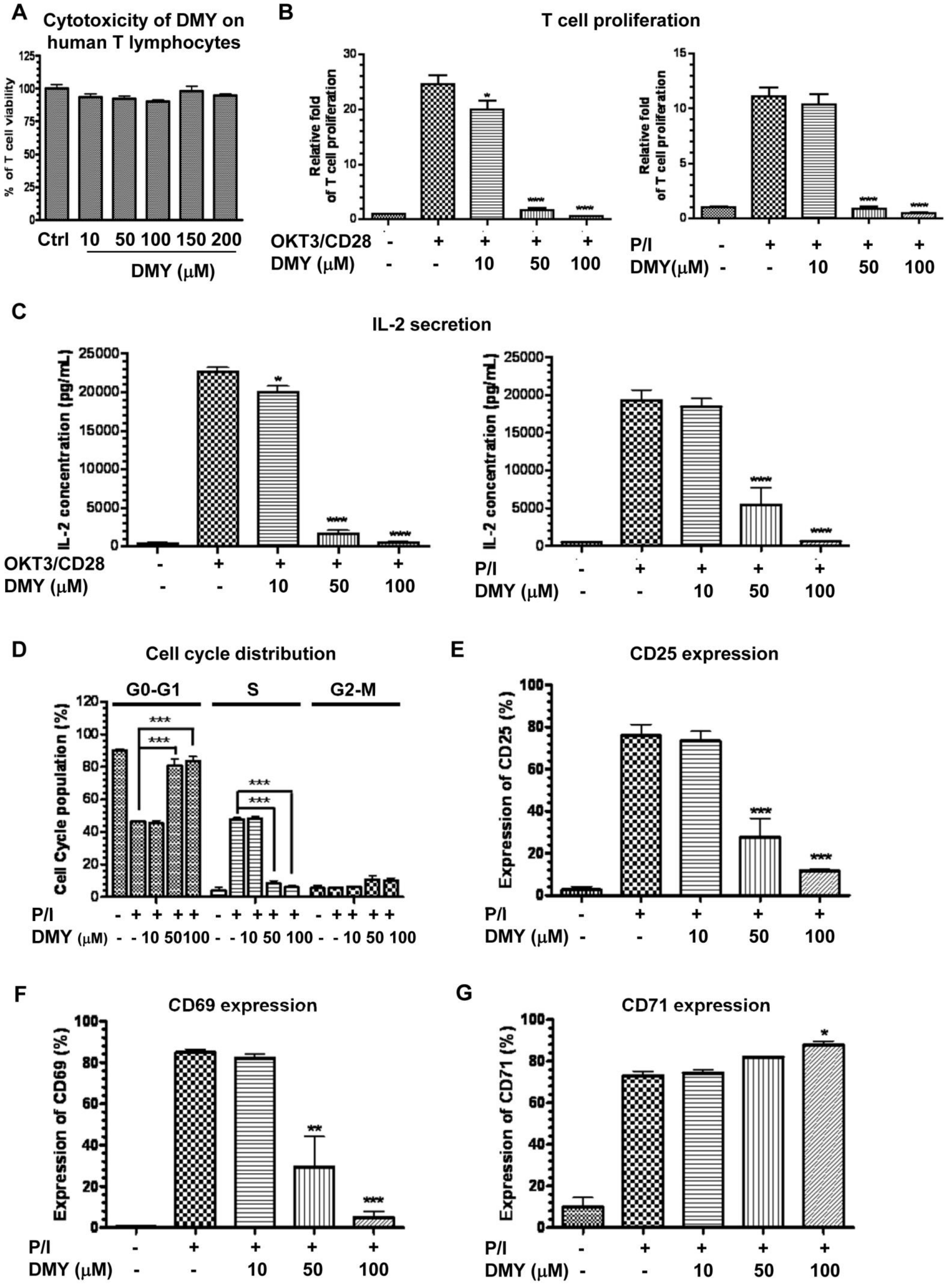
A
Cytotoxicity of DMY on human T lymphocytes
% of T cell viability
125
100
75
50
25
0
Ctrl 10 50 100 150 200
DMY (μM)
B
T cell proliferation
Relative fold of T cell proliferation
30
20
10
0
OKT3/CD28 - + + + +
DMY (μM) - - 10 50 100
15
10
5
0
P/I - + + + +
DMY(μM) - - 10 50 100
C
IL-2 secretion
IL-2 concentration (pg/mL)
25000
20000
15000
10000
5000
0
OKT3/CD28 - + + + +
DMY (μM) - - 10 50 100
P/I - + + + +
DMY (μM) - - 10 50 100
D
Cell cycle distribution
G0-G1
S
G2-M
Cell Cycle population (%)
120
100
80
60
40
20
0
***
P/I - + + + + - + + + + - + + + +
DMY (μM) - - 10 50 100 - - 10 50 100 - - 10 50 100
E
CD25 expression
Expression of CD25 (%)
100
80
60
40
20
0
***
P/I - + + + +
DMY (μM) - - 10 50 100
F
CD69 expression
Expression of CD69 (%)
100
80
60
40
20
0
**
***
P/I - + + + +
DMY (μM) - - 10 50 100
G
CD71 expression
Expression of CD71 (%)
100
80
60
40
20
0
*
P/I - + + + +
DMY (μM) - - 10 50 100

Fig. S2. DMY inhibited IKK-β–NF-κB signalling and in turn suppressed human T cell activation and T cell surface marker expression.

(A) Cytotoxicity of DMY on T lymphocytes. (B) DMY inhibited OKT-3/CD28- or P/I-induced human T cell proliferation. (C) DMY inhibited OKT-3/CD28- or P/I-induced IL-2 cytokine secretion. The isolated human T lymphocytes were co-stimulated with OKT-3/CD28 or P/I in the presence of the indicated concentrations of DMY. T cell proliferation was then measured by BrdU proliferation assay. For IL-2 cytokine measurement, the cell-free culture supernatants were harvested for IL-2 ELISA. (D) DMY arrested P/I-mediated cell cycle progression in isolated human T cells. Human T lymphocytes pretreated with the indicated concentrations of DMY and stimulated with or without P/I for 72 h. The cells were then fixed and stained with propidium iodide for cell cycle analysis. (E) DMY suppressed the P/I-mediated expression of the IL-2 receptor, CD25. (F) DMY suppressed P/I-mediated expression of the early-stage T cell activation marker CD69. (G) DMY slightly increased P/I-mediated expression of the late-stage T cell activation marker CD71. The isolated human T cells pretreated with the indicated concentrations of DMY were co-stimulated with P/I. The cells were then stained with specific antibodies in the combination of CD3-PE with CD25-FITC or CD69-FITC or CD71-FITC for 30 min. The cell surface expression of CD25, CD69 and CD71 antigens were then analysed by flow cytometry. Data in each column represented the mean ± SEM of three independent experiments. *$P<0.05$, **$P<0.01$, ***$P<0.001$.

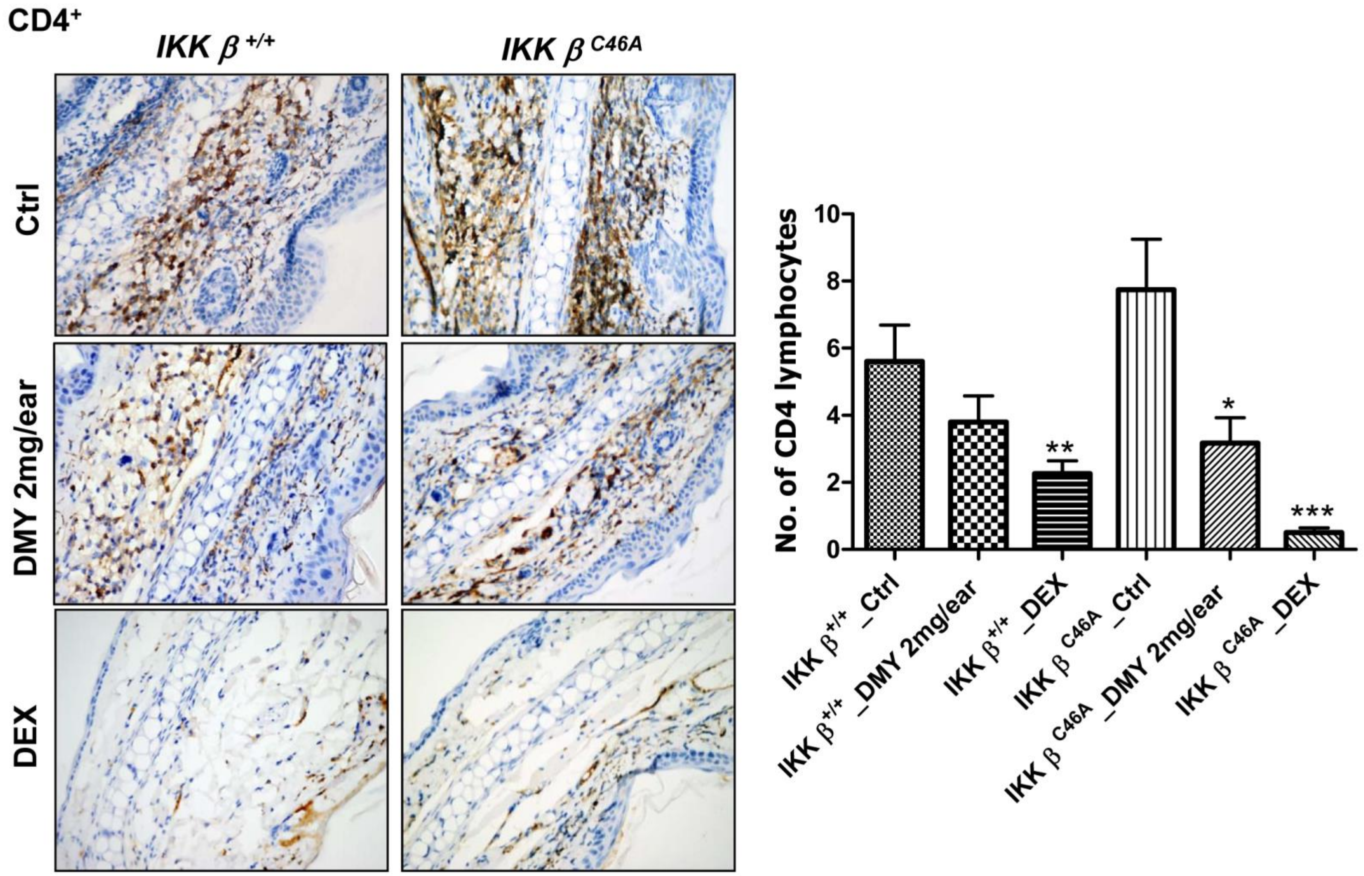

Fig. S3. DMY treatment decreased the population of CD4$^+$ T lymphocytes in the ear tissues of DTH-*IKK-β* $^{+/+}$ and -*IKK-β* ^{C46A} mice. The CD4$^+$ T cells were quantitated by immunohistochemical analysis of the ear sections from DTH mice. Bar charts represented the average number of CD4$^+$ T lymphocytes found in the ear sections of both wild-type and mutant DTH animals.
